# A 92 protein inflammation panel performed on sonicate fluid differentiates periprosthetic joint infection from non-infectious causes of arthroplasty failure

**DOI:** 10.1038/s41598-022-20444-9

**Published:** 2022-09-27

**Authors:** Cody R. Fisher, Harold I. Salmons, Jay Mandrekar, Kerryl E. Greenwood-Quaintance, Matthew P. Abdel, Robin Patel

**Affiliations:** 1 Department of Immunology, Mayo Clinic Graduate School of Biomedical Sciences, Mayo Clinic, Rochester, MN USA; 2 Division of Clinical Microbiology, Department of Laboratory Medicine and Pathology, Mayo Clinic, 200 First Street SW, Rochester, MN 55905 USA; 3 Department of Orthopedic Surgery, Mayo Clinic, Rochester, MN USA; 4 Department of Quantitative Sciences, Mayo Clinic, Rochester, MN USA; 5 Division of Public Health, Infectious Diseases and Occupational Medicine, Department of Medicine, Mayo Clinic, Rochester, MN USA

**Keywords:** Infectious-disease diagnostics, Antimicrobial responses, Proteomics

## Abstract

Periprosthetic joint infection (PJI) is a major complication of total joint arthroplasty, typically necessitating surgical intervention and prolonged antimicrobial therapy. Currently, there is no perfect assay for PJI diagnosis. Proteomic profiling of sonicate fluid has the potential to differentiate PJI from non-infectious arthroplasty failure (NIAF) and possibly clinical subsets of PJI and/or NIAF. In this study, 200 sonicate fluid samples, including 90 from subjects with NIAF (23 aseptic loosening, 35 instability, 10 stiffness, five osteolysis, and 17 other) and 110 from subjects with PJI (40 *Staphylococcus aureus*, 40 *Staphylococcus epidermidis*, 10 *Staphylococcus lugdunensis*, 10 *Streptococcus agalactiae*, and 10 *Enterococcus faecalis*) were analyzed by proximity extension assay using the 92 protein Inflammation Panel from Olink Proteomics. Thirty-seven of the 92 proteins examined, including CCL20, OSM, EN-RAGE, IL8, and IL6, were differentially expressed in PJI versus NIAF sonicate fluid samples, with none of the 92 proteins differentially expressed between staphylococcal versus non-staphylococcal PJI, nor between the different types of NIAF studied. IL-17A and CCL11 were differentially expressed between PJI caused by different bacterial species, with IL-17A detected at higher levels in *S. aureus* compared to *S. epidermidis* and *S. lugdunensis* PJI, and CCL11 detected at higher levels in *S. epidermidis* compared to *S. aureus* and *S. agalactiae* PJI. Receiver operative characteristic curve analysis identified individual proteins and combinations of proteins that could differentiate PJI from NIAF. Overall, proteomic profiling using this small protein panel was able to differentiate between PJI and NIAF sonicate samples and provide a better understanding of the immune response during arthroplasty failure.

## Introduction

Periprosthetic joint infection (PJI) occurs in 1–2% of patients undergoing total joint arthroplasty^1–3^. While relatively rare, PJI results in significant time and expense for the healthcare industry and affected individuals^4–6^. PJI is primarily caused by biofilm-producing bacteria, such as *Staphylococcus aureus*, *Staphylococcus epidermidis*, *Staphylococcus lugdunensis, Streptococcus agalactiae*, and *Enterococcus faecalis*^1,7,8^. These bacteria produce immune modulating products and grow as robust biofilms on prosthetic devices, where they evade antimicrobial treatments and the host immune response^2,9,10^. In addition to antimicrobial treatment, surgery is usually indicated for PJI, with surgery-type guided by acuity of infection and other factors. For acute PJI, irrigation and debridement with component retention followed by long-term antibiotics is advocated. For non-acute cases, alongside antibiotic treatment, resection of components is typically necessary, with two-stage exchange arthroplasties frequently used in the United States^1,11–13^. Non-infectious arthroplasty failure (NIAF) is another arthroplasty-associated complication that can lead to a need for surgical revision. There are a variety of NIAF causes, including aseptic loosening, instability, osteolysis, and stiffness, among others^14–17^. It may be difficult to clinically differentiate between NIAF and PJI due to the low quantitative burden and virulence of some infecting pathogens^18^.

There is no perfect diagnostic approach for PJI, making fast and accurate determination of infection a clinical challenge in some cases^19^. Traditional bacterial culture strategies may be limited by insufficient amounts of sample collected and have the potential for growth of organisms that may be contaminants or pathogens, or negative culture growth altogether. To address these limitations, diagnostic strategies in identifying PJI have incorporated host-derived characteristics to identify infection. These characteristics, including erythrocyte sedimentation rate, C-reactive protein, and D-dimer measurement in blood; nucleated cell count, neutrophil percentage, C-reactive protein and alpha-defensin^20–22^ in synovial fluid; and intraoperative tissue histology and purulence, can provide evidence of underlying infection^1,11^. Even so, determining the presence of infection remains challenging in some cases, and once defined, it is helpful to define the microbial etiology to direct treatment. Recently, molecular and multi-omics approaches have been identified as potential avenues for finding candidate biomarkers and developing immune profiles to aid in diagnostic and treatment decisions for infected and non-infectious arthroplasty revision patients^23,24^.

For example, various synovial fluid, serum, and periprosthetic tissues proteins have previously been described as PJI differentiating biomarkers, including calprotectin, lipocalin-2, and lactotransferrin, among others^21,22,25–30^. Metagenomic and transcriptomic analyses have been conducted on PJI and NIAF sonicate fluids, biomaterials sonicated and collected from excised implanted devices after arthroplasty revision surgery^31^, though the proteomic composition of sonicate fluid has not yet been established^24,32–34^. Unlike the indirect (DNA and RNA) functional response profiles constructed from metagenomic or transcriptomic approaches, proteomic analysis provides a direct (protein) functional host response profile of the PJI or NIAF joint microenvironment.

The Olink Proteomics - Inflammation Panel is a well described and applied platform that uses proximal extension assay (PEA) to quantify 92 target proteins (Supplemental Fig. 1)^35^. Recently, the panel has been utilized to characterize the inflammatory response during infectious diseases, including leishmaniasis, tuberculosis, urinary tract, HIV, and COVID-19^36–40^. The panel has also been used to investigate musculoskeletal inflammation during rheumatoid arthritis and in a porcine model of *S. aureus* bone infection^41–43^. PJI and NIAF proteomic profiling using this proteomic platform may give direct evidence towards potential PJI versus NIAF diagnostic biomarkers, avenues for novel treatment options, and provide additional illumination of the inflammatory response during PJI.

In this study, the 92 protein Inflammation Panel from Olink Proteomics (Uppsala, Sweden) was performed on sonicate fluid collected from 200 uninfected or PJI individuals with arthroplasty failure to determine whether this panel can be used to define infection status, determine causative bacterial species in PJI, or define underlying causes of arthroplasty failure in NIAF.

## Methods

### Statement of ethical approval

This was a retrospective study of sonicate fluids remaining after clinical testing and was approved by the Mayo Clinic Institutional Review Board with a waiver of informed consent (09-000,808—“Detection of Biofilms on Explanted Orthopedic Devices”). The study was performed is in accordance with relevant guidelines and regulations.

### Clinical samples and study design

Sonicate fluids collected between July 2007 and August 2018 from 200 revision total hip or knee arthroplasties were studied. Arthroplasty components had been removed and subjected to sonication for clinical purposes; briefly, removed total hip or knee arthroplasty components had been placed in Ringer’s solution and subjected to vortexing and sonication, as described in^31^. Samples were frozen and stored at − 80 °C until used. Ninety of the samples were harvested from uninfected individuals and 110 were from individuals with PJI as determined by the classification scheme published by Parvizi et al.^44^. The 90 uninfected samples were further separated into subgroups, including 23 aseptic loosening, 35 instability, 10 stiffness, five osteolysis, and 17 other. Indications for failure were retrospectively reviewed by a board-certified orthopedic surgeon, with the single best fitting diagnosis selected based on review of the electronic medical records, in concert with the diagnosis of the original orthopedic surgeon^45–51^. In situations with multiple potential failure modes, the single most likely to have been the inciting event leading to revision was selected for the purpose of analysis. Further definitions of each NIAF subtype can be found in Table S1. Of the 110 PJI samples, 40 were infected with *S. aureus*, 40 *S. epidermidis*, 10 *S. lugdunensis*, 10 *S. agalactiae*, and 10 *E. faecalis*. Patients with indicated underlying inflammatory or neoplastic disorders were excluded.

### Proteomic profiling

Sonicate fluids were thawed, and total protein concentrations of each determined by the Coomassie (Bradford) Protein Assay Kit (23,200, Thermo Scientific, Rockford, lL)^52^. Samples were diluted to 700 µg/ml total protein concentration in 0.9% sodium chloride (2F7122, Baxter, Deerfield, IL) and 50 µl aliquots of each sample randomized into wells of twin.tec skirted 96 well PCR plates (951,020,460, Eppendorf, Hamburg, Germany). Samples were frozen on dry ice and sent to Olink Proteomics (Uppsala, Sweden), where protein quantification was performed using PEA with the Olink Proteomics - Inflammation Panel, including 92 target proteins (Supplemental Fig. 1)^35^. Olink Proteomics uses Normalized Protein eXpression (NPX), an arbitrary log_2_ value, for protein normalization and quantification.

### Bioinformatics and statistical analysis

Data was analyzed, organized, and graphed in RStudio v1.2.5042^53^ using R-packages, including “factoextra”^54^ for principal component analysis (PCA) and contribution plots, “ComplexHeatmap”^55^ for heatmap creation, “umap”^56^ for umap creation, “plotROC”^57^ and “ggplot2”^58^ for receiver operating characteristic (ROC) curve creation, and “OlinkAnalyze” (https://github.com/Olink-Proteomics/OlinkRPackage) for volcano plot creation. Combinational area under the ROC curves were estimated using univariable and multivariable logistic regression models. This analysis was performed using SAS software version 9.4 (SAS Inc., Cary, NC, USA). NPX counts were analyzed and graphed in Graphpad Prism 9 v9.2.0 (San Diego, CA, USA). Statistical significance was determined via Welch’s 2-sample t-test, with Benjamini–Hochberg multiple comparison adjustment.

### Ethics approval and consent to participate

The study was approved by the Mayo Clinic Institutional Review Board (#09-000,808).

## Results

In total, sonicate fluid samples from 200 subjects, including 90 who had NIAF (23 aseptic loosening, 35 instability, 10 stiffness, five osteolysis, and 17 other) and 110 who had PJI (40 *S. aureus*, 40 *S. epidermidis*, 10 *S. lugdunensis*, 10 *S. agalactiae*, and 10 *E. faecalis* PJIs) were analyzed via PEA using the Olink Proteomics - Inflammation Panel (Table 1), which includes 92 proteins (Supplemental Table 1).

**Table 1:** Sonicate fluid samples studied.

Sample group	Sample size (n)	Mean age (years)	Sex (% male)
Non-infectious arthroplasty failure	90	66	42
Aseptic loosening	23	69	39
Instability	35	63	46
Stiffness	10	69	40
Osteolysis	5	63	20
Other	17	68	47
Periprosthetic joint infection	110	66	52
* Staphylococcus aureus*	40	68	55
* Staphylococcus epidermidis*	40	64	50
* Staphylococcus lugdunensis*	10	68	50
* Enterococcus faecalis*	10	67	50
* Streptococcus agalactiae*	10	62	60
Total	200	66	48

### Profiling of PJI versus NIAF samples

**Figure 1:**
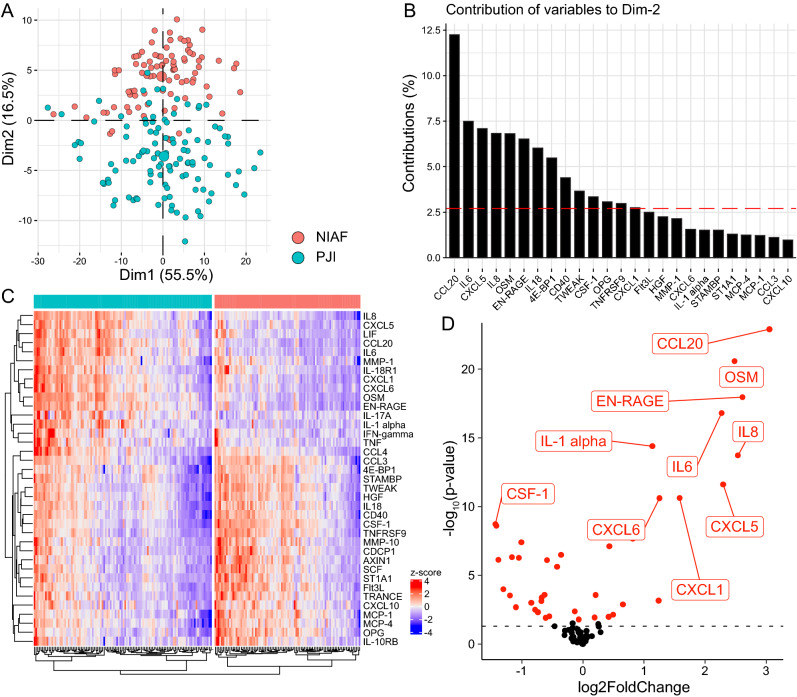
Proteomic profiling of sonicate fluids from periprosthetic joint infection (PJI) and non-infectious arthroplasty failure (NIAF) using the Olink Proteomics - Inflammation Panel. (**A**) Principal component analysis (PCA) showing grouping of NIAF and PJI based on Dim1 and Dim2. Larger points indicate geometric means of the sample population. (**B**) Contribution plot showing protein targets with the most contribution to separation on the PCA Dim2. (**C**) Heatmap analysis of protein expression z-scores in NIAF and PJI samples, with differentially expressed protein targets clustered and shown. (**D**) Volcano plot of NIAF versus PJI samples, with significantly and non-significantly differentially expressed protein targets shown in red and black, respectively. The top 10 differentially expressed proteins are labeled. The horizontal dashed line in D designates a *P*-value = 0.05. Statistical significance was determined via Welch’s 2-sample t-test, with Benjamini–Hochberg multiple comparison adjustment. Data depicted are for NIAF (n = 90) and PJI (n = 110) sonicate fluid samples.

Comparative analysis was conducted to determine whether PJI and NIAF could be differentiated based on sonicate fluid analysis using the Olink Proteomics  - Inflammation Panel (Fig. 1). PCA plotting was able to differentiate infected and uninfected sample clusters along dimension 2 (Dim2), though only accounting for 16.5% of variation within the full panel results (Fig. 1A). Proteins contributing most to separation in PCA Dim2 were CCL20, IL6, CXCL5, IL8, and OSM, with contributions percentages of approximately 12, 7.5, 7, 6.5, and 6.5%, respectively (Fig. 1B). To further determine which proteins were important for PJI versus NIAF sample differentiation, comparative statistical analyses were conducted on individual target proteins. The expression of 37 of the 92 target proteins examined was significantly different when comparing PJI to NIAF samples, with adjusted *P*-values <0.05 using Welch’s 2-sample t-test, with Benjamini–Hochberg multiple comparison adjustment (Supplemental Table 2). PJI and NIAF samples were also able to be separated by protein expression using heatmap analysis (Fig. 1C). Sixteen proteins, including CCL20, OSM, EN-RAGE, IL8, and IL6, were present at higher levels in PJI compared to NIAF samples, with Log_2_FoldChange values of 3.05, 2.48, 2.61, 2.54, and 2.27, respectively, while 21, including CSF-1, OPG, MCP-1, and 4E-BP1, were present at lower levels in PJI compared to NIAF samples, with Log_2_FoldChange values of − 1.43, − 1.42, − 1.39, and − 1.30, respectively (Fig. 1D).

**Figure 2:**
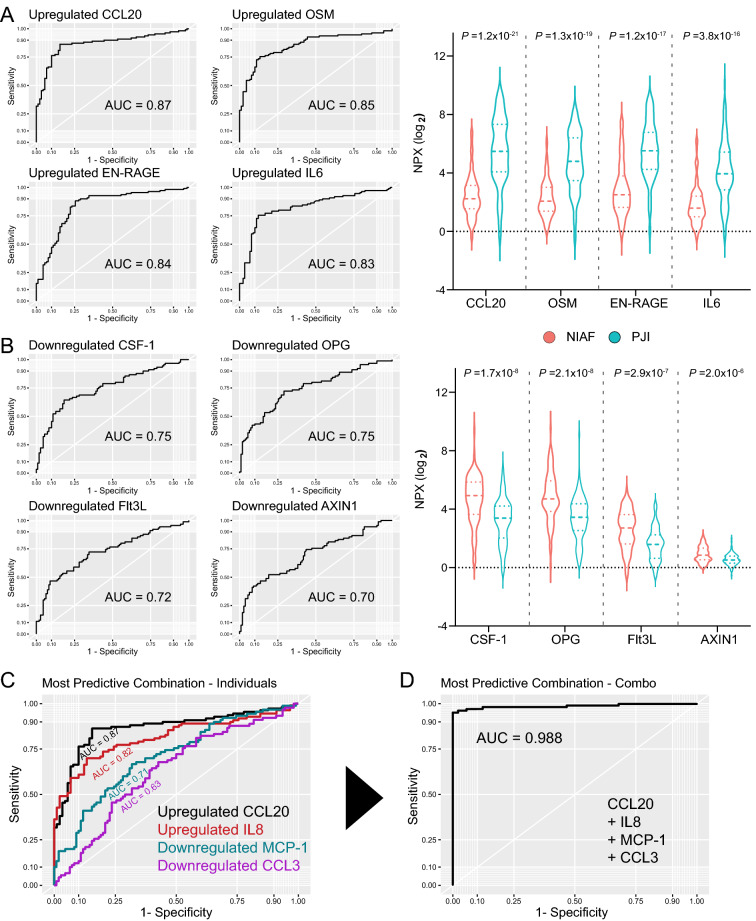
Top predictive proteins differentiating periprosthetic joint infection (PJI) from non-infectious arthroplasty failure (NIAF) identified in sonicate fluid using the Olink Proteomics - Inflammation Panel. Receiver operative characteristic (ROC) curves, including area under the curve (AUC), and normalized protein expression (NPX) counts, including associated adjusted *P*-values, of the top four (**A**) upregulated and (**B**) downregulated proteins in PJI compared to NIAF are shown. ROC curves showing the most differentially predictive combination of protein targets, including (**C**) individual and (**D**) combined protein target analyses. Statistical significance was determined via Welch’s 2-sample t-test, with Benjamini–Hochberg multiple comparison adjustment. Data depicted are for NIAF (n = 90) and PJI (n = 110) sonicate fluid samples.

ROC curves were created to further evaluate the potential utility of identifying these proteins in distinguishing PJI from NIAF (Fig. 2). The proteins expressed at higher levels in PJI compared to NIAF by this analysis were CCL20, OSM, EN-RAGE, and IL6, with area under the curve (AUC) values of 0.87, 0.85, 0.84, and 0.83, respectively (Fig. 2A). The proteins expressed at lower levels in PJI compared to NIAF by this analysis were CSF-1, OPG, Flt3L, and AXIN1, with AUC values of 0.75, 0.75, 0.72, and 0.70, respectively (Fig. 2B). Analysis of target protein combinations most predictive of PJI were also assessed, finding that the combination of higher CCL20, higher IL8, lower MCP-1, and lower CCL3, with individual AUC values of 0.87, 0.82, 0.71, 0.63, respectively, resulted in the highest PJI predictive score, with a combinational AUC value of 0.988 (Fig. 2C).

### Profiling of staphylococcal versus non-staphylococcal and of species versus species PJI

**Figure 3:**
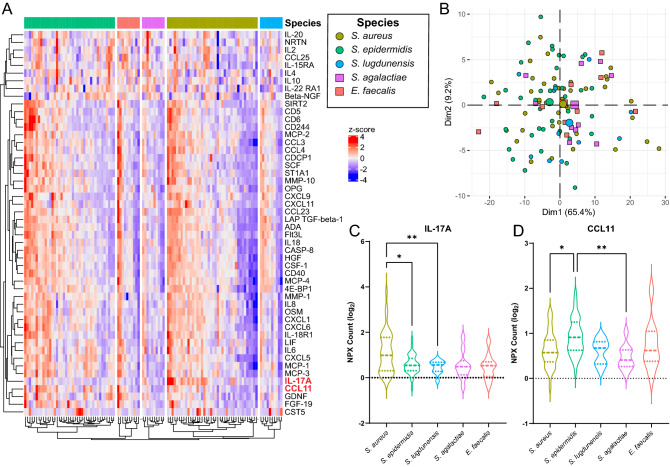
Analysis of staphylococcal versus non-staphylococcal and species versus species periprosthetic joint infection (PJI) using the Olink Proteomics Inflammation Panel. (**A**) Heatmap analysis of protein expression z-scores in each PJI-causing bacterial species examined, with the 50 most statistically significant protein targets clustered and shown. IL-17A and CCL11 (highlighted in red) were the only protein targets statistically differentially expressed in any species versus species PJI-causing bacterial species pairing. (**B**) Principal component analysis (PCA) showing grouping of staphylococcal (circles) versus non-staphylococcal (square) PJI and species versus species PJI-causing bacterial species (indicated by colors) based on Dim1 and Dim2. Larger points indicate geometric means of the sample population. Normalized protein expression (NPX) counts of (**C**) IL-17A and (**D**) CCL11 in species versus species PJI-causing bacterial species. Statistical significance was determined via Welch’s 2-sample t-test, with Benjamini–Hochberg multiple comparison adjustment (*P ≤ 0.05; **P ≤ 0.01). Data depicted are for staphylococcal (n = 90) and non-staphylococcal (n = 20) PJI sonicate fluid samples, separated into *Staphylococcus aureus* (n = 40), *Staphylococcus epidermidis* (n = 40), *Staphylococcus lugdunensis* (n = 10), *Streptococcus agalactiae* (n = 10), and *Enterococcus faecalis* (n = 10) PJI.

Comparative analyses were conducted between staphylococcal versus non-staphylococcal, and of species versus species PJI, including PJI caused by *S. aureus*, *S. epidermidis*, *S. lugdunensis*, *S. agalactiae*, and *E. faecalis* (Fig. 3). Staphylococcal versus non-staphylococcal PJI subgroups were not differentiated by heatmap analysis based on individual protein expression profiles (Fig. 3A), or PCA based on full panel expression (Fig. 3B). Of the 92 proteins examined, none were detected at different levels in staphylococcal versus non-staphylococcal samples (Supplemental Fig. 1).

Though overall profile differentiation of species versus species PJI-causing bacterial species was not clear via PCA (Fig. 3B), IL-17A and CCL11, were differentially expressed between groups (Fig. 3A). IL-17A was significantly increased in *S. aureus* compared to *S. epidermidis* (*P* ≤ 0.05) and *S. lugdunensis* (*P* ≤ 0.01) PJI, with mean log_2_ NPX counts of 1.11 compared to 0.57 and 0.47, respectively (Fig. 3C). CCL11 was significantly increased in *S. epidermidis* compared to *S. aureus* (*P* ≤ 0.05) and *S. agalactiae* (*P* ≤ 0.01) PJI, with mean log_2_ NPX counts of 0.92 compared to 0.64 and 0.46, respectively (Fig. 3D).

### Analysis of NIAF subtypes

**Figure 4:**
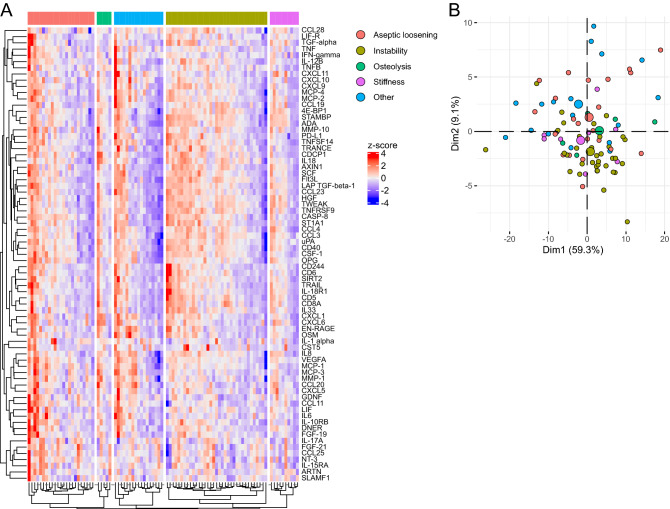
Proteomic profiling of non-infectious arthroplasty failure (NIAF) subtypes using the Olink Proteomics - Inflammation Panel. (**A**) Heatmap analysis of NIAF subset protein expression z-scores, including aseptic loosening, instability, osteolysis, stiffness, and other, with protein targets clustered. (**B**) Principal component analysis (PCA) showing grouping of NIAF based on Dim1 and Dim2. Larger points indicate geometric means of the sample population. Statistical significance was determined via Welch’s 2-sample t-test, with Benjamini–Hochberg multiple comparison adjustment. Data depict aseptic loosening (n = 23), instability (n = 35), osteolysis (n = 10), stiffness (n = 5), and other (n = 17) sonicate fluid samples.

Comparative analyses of the NIAF subgroups, including aseptic loosening, instability, osteolysis, stiffness, and other, were conducted to determine whether they could be differentiated using this technique (Fig. 4). Subgroups were not able to be separated based on individual protein targets via heatmap analysis; none the 92 protein targets were present at significantly different levels (Fig. 4A). PCA was also unable to separate NIAF subgroups based on the panel of 92 proteins (Fig. 4B).

## Discussion

In this study, the proteomic profile of 200 sonicate fluid samples collected from prostheses after revision arthroplasty was evaluated. Of the 200 samples analyzed, 90 were from subjects with NIAF and 110 from those with PJI. The NIAF samples were further broken down to subgroups including 23 aseptic loosening, 35 instability, 10 stiffness, five osteolysis, and 17 other causes. The PJI samples were further broken down into 40 *S. aureus*, 40 *S. epidermidis*, 10 *S. lugdunensis*, 10 *E. faecalis*, and 10 *S. agalactiae* PJIs. Profiles were investigated using the PEA Inflammation Panel offered by Olink Proteomics, where the expression of 92 target proteins was quantified for each sonicate fluid sample.

Proteomic profiles were created to compare PJI and NIAF sonicate fluids. PCA was able to separate PJI from NIAF samples along Dim2, though accounting for less than 20% of the overall variability in the sample set. CCL20, IL6, CXCL5, IL8, and OSM were top contributors to the group separation. In all, 37 proteins were found at significantly different levels between PJI and NIAF, with 16 proteins found at higher levels in PJI compared to NIAF, and 21 found at lower levels in PJI compared to NIAF. Transcriptomic analysis on 40 of the 90 NIAF samples and 32 of the 110 PJI samples (8 *S. aureus*, 15 *S. epidermidis*, 5 *S. lugdunensis*, and 4 *S. agalactiae*) used in this study has been recently reported^34^. Of the 37 differentially abundant proteins, 25 were found have differentially expressed transcripts via RNA-sequencing. In every recapitulated case, whether the target was up- or downregulated in PJI compared to NIAF remained consistent between proteomic and transcriptomic analysis. These results give further evidence and validation towards valuable use of sonicate fluid as a differentiable specimen of PJI and NIAF.

Using ROC analyses, CCL20, OSM, IL6, and EN-RAGE were identified as more predictive of PJI compared to NIAF (AUC > 0.80) and CSF-1, OPG, Flt3L, and AXIN1 were more predictive of NIAF compared to PJI (AUC ≥ 0.70). An AUC value of 1.0 is considered perfect, with 100% specificity and sensitivity^59,60^. CCL20 (also known as macrophage inflammatory protein 3α, MIP-3α) was most differentially increased in PJI compared to NIAF. CCL20 is a chemokine implicated in a wide range of inflammatory responses, such as those during airway irritation, irritable bowel disease, psoriasis, cancer, and neuroinflammation^61–65^. CCL20 is chemoattractant for leukocytes, including neutrophils and dendritic cells, and induces a proinflammatory phenotype in macrophages through CCR6 receptor signalling^66^. CCL20 also has antimicrobial activity, with similar properties with host β-defensins^67–69^. OSM, or oncostatin M, and IL6 are members of the gp130 family of cytokines and have several pro- and anti-inflammatory functions^70,71^. Of note, both OSM and IL6 are directly involved in bone homeostasis by osteoblasts and osteoclasts and in the inflammatory response towards infection^72–74^. Like IL6, EN-RAGE is produced by macrophages and neutrophils as an antimicrobial response to infection or inflammation^75,76^. Each of these proteins, among others, was found at higher levels in PJI compared to NIAF, rendering them potential candidates as diagnostic biomarkers and novel treatment targets.

Interestingly, CSF-1 (also known as macrophage colony-stimulating factor [M-CSF]), OPG (or osteoprotegerin), Flt3L (or FMS-like tyrosine kinase 3 ligand) and AXIN1 were most significantly differentially decreased in PJI compared to NIAF. These proteins primarily have inflammatory and/or bone homeostatic roles. CSF-1 is involved in the differentiation and activation of macrophages, stimulating chemotaxis and phagocytosis^77,78^, while Flt3L is needed for dendritic cell development and hematopoietic progenitor activation^79,80^. CSF-1, OPG, and AXIN1 are important for bone homeostasis, where CSF-1 is released by osteoblasts to directly induce osteoclastogenesis^81^. In contrast, OPG suppresses osteoclastogenesis, and its expression is upregulated by osteoblasts through β-catenin signalling^82^, which has recently been shown to be inhibited by AXIN1^83^. The exact mechanisms for the downregulation of these proteins, their interactions, and how they impact host response to PJI is unknown and warrants further study.

ROC curve analyses were also conducted to determine the most predictive combination of protein targets able to separate PJI from NIAF. This analysis found that the combination of CCL20, IL8, MCP-1, and CCL3 resulted in an AUC value of 0.988, most predictive of all protein combinations. Interestingly, while each of these proteins is important for chemoattraction and inflammatory response of macrophages and neutrophils^66,84–86^, CCL20, as mentioned previously, and IL8 were present in higher levels in PJI compared to NIAF, while MCP-1 and CCL3 were present in lower levels in PJI compared to NIAF. While some identified proteins, including IL6, IL8, IL17A, IFNγ, and TWEAK have been found in synovial fluid or serum of subjects with PJI^22,27,30^, others, such as CCL20, OSM, EN-RAGE, and CXCL6, are more novel. Further investigation into the diagnostic power of these candidate biomarkers and potential for targeted treatment is needed.

Analysis was also performed to determine whether subsets of PJI could be differentiated using the Olink Proteomics - Inflammation Panel. Only IL-17A and CCL11 had differential abundance when comparing different species of bacteria causing PJI. IL-17A was found at higher levels in *S. aureus* compared to *S. epidermidis* and *S. lugdunensis* PJI and CCL11 at higher levels in *S. epidermidis* compared to *S. aureus* and *S. agalactiae* PJI. Both IL-17A and CCL11 are immune activators and stimulators of bone resorption via osteoclastogenesis^87,88^. No proteins were differentially expressed in staphylococcal versus non-staphylococcal samples. Unlike the PJI versus NIAF analyses, staphylococcal versus non-staphylococcal, or species versus species PJI-causing bacterial groups were not able to be differentiated by PCA, or heatmap analysis. Similarity of the infected proteomic profiles suggests that the inflammatory response may be comparable across PJI caused by different species, at least as determined with the Olink Proteomics - Inflammation Panel, with possible evidence of differential amounts of IL-17A and CCL11 between some PJI caused by some species of *Staphylococcus.*

Being able to differentiate underlying causes of NIAF may provide opportunities for early diagnosis and potentially clinical intervention. Unfortunately, analysis of NIAF subsets, including aseptic loosening, instability, stiffness, osteolysis, or other, did not identify significantly different protein expression across any of the NIAF subsets. Further, these were not separable by heatmap analysis or by PCA. This may be attributable to the nature and function of the proteins under study as well as the proteomic heterogeneity of aseptic failure samples analyzed in this study. Predictive assays to determine the causes of NIAF may have clinical benefit, so additional target proteins outside of those evaluated here should be examined in future studies.

There are several limitations related to this study. Firstly, sonicate fluid samples studied were originally harvested, processed, and stored using protocols to preserve bacteria. The sonication and/or storage methods used may have differentially influenced overall or individual host protein concentrations, impacting downstream proteomic expression analysis. Additionally, the proteomic analysis was limited to the predetermined 92 proteins included in the Olink Proteomics - Inflammation Panel. Other proteins important for proteomic profiling and possible differentiation of PJI and NIAF were likely not included in this analysis. Next, those with underlying inflammatory or neoplastic disorders were excluded from this study; therefore, the results may not be applicable to those with underlying inflammatory or neoplastic disorders. Lastly, while the overall sonicate fluid sample size was 200, only five species of PJI-causing bacteria were analyzed and some sample subsets were limited in size, with only 10 *S. lugdunensis*, 10 *S. agalactiae*, and 10 *E. faecalis* PJI cases, and only 10 stiffness and five osteolysis NIAF cases studied. Continued study to bolster the sample cohort and confirm the results are warranted.

## Conclusions

Overall, results from this study showed that the Olink Proteomics - Inflammation Panel performed on sonicate fluid differentiated between PJI and NIAF, but not between staphylococcal versus non-staphylococcal PJI, PJI caused by different bacterial species (except for some *Staphylococcus* species) or causes of NIAF. These findings provide additional characterization of the immune response mounted against PJI during arthroplasty failure. Further proteomic analysis in additional sample types and using unbiased quantitative techniques are necessary.

## Supplementary Information

Supplementary Information.

## Data Availability

The datasets used and/or analyzed during the current study are available from the corresponding authors on reasonable request.
