# Author Correction: A 92 protein inflammation panel performed on sonicate fluid differentiates periprosthetic joint infection from non-infectious causes of arthroplasty failure

**DOI:** 10.1038/s41598-023-31836-w

**Published:** 2023-03-22

**Authors:** Cody R. Fisher, Harold I. Salmons, Jay Mandrekar, Kerryl E. Greenwood-Quaintance, Matthew P. Abdel, Robin Patel

**Affiliations:** 1 Department of Immunology, Mayo Clinic Graduate School of Biomedical Sciences, Mayo Clinic, Rochester, MN USA; 2 Division of Clinical Microbiology, Department of Laboratory Medicine and Pathology, Mayo Clinic, 200 First Street SW, Rochester, MN 55905 USA; 3 Department of Orthopedic Surgery, Mayo Clinic, Rochester, MN USA; 4 Department of Quantitative Sciences, Mayo Clinic, Rochester, MN USA; 5 Division of Public Health, Infectious Diseases and Occupational Medicine, Department of Medicine, Mayo Clinic, Rochester, MN USA

Correction to: *Scientific Reports* 10.1038/s41598-022-20444-9, published online 27 September 2022

The original version of this Article contained errors in the numbers of upregulated and downregulated proteins.

As a result, in the Results section, under the subheading ‘Profiling of PJI versus NIAF samples’,

“Fifteen proteins, including CCL20, OSM, EN-RAGE, IL8, and IL6, were present at higher levels in PJI compared to NIAF samples, with Log_2_FoldChange values of 3.05, 2.48, 2.61, 2.54, and 2.27, respectively, while 22, including CSF-1, OPG, MCP-1, and 4E-BP1, were present at lower levels in PJI compared to NIAF samples, with Log_2_FoldChange values of − 1.43, − 1.42, − 1.39, and − 1.30, respectively (Fig. 1D).”

now reads:

“Sixteen proteins, including CCL20, OSM, EN-RAGE, IL8, and IL6, were present at higher levels in PJI compared to NIAF samples, with Log_2_FoldChange values of 3.05, 2.48, 2.61, 2.54, and 2.27, respectively, while 21, including CSF-1, OPG, MCP-1, and 4E-BP1, were present at lower levels in PJI compared to NIAF samples, with Log_2_FoldChange values of − 1.43, − 1.42, − 1.39, and − 1.30, respectively (Fig. 1D).”

In addition, in the Discussion section,

“In all, 37 proteins were found at significantly different levels between PJI and NIAF, with 15 proteins found at higher levels in PJI compared to NIAF, and 22 found at lower levels in PJI compared to NIAF.”

now reads:

“In all, 37 proteins were found at significantly different levels between PJI and NIAF, with 16 proteins found at higher levels in PJI compared to NIAF, and 21 found at lower levels in PJI compared to NIAF.”

The original Article has been corrected.

