# Supplementary material for: A 92 protein inflammation panel performed on sonicate fluid differentiates periprosthetic joint infection from non-infectious causes of arthroplasty failure
Source: Sci Rep. 2022 Sep 27;12:16135. doi: 10.1038/s41598-022-20444-9 (PMC9514711; doi:10.1038/s41598-022-20444-9)
Supplement: Supplementary file 1 — Supplementary Information. [file 41598_2022_20444_MOESM1_ESM.pdf]

A 92 Protein Inflammation Panel Performed on Sonicate Fluid Differentiates Periprosthetic Joint Infection from Non-Infectious Causes of Arthroplasty Failure

Cody R. Fisher[a,b], Harold I. Salmons[c], Jay Mandrekar[b,d], Kerryl E. Greenwood-Quaintance[b], Matthew P. Abdel[c], Robin Patel[b,e]#

[a]Mayo Clinic Graduate School of Biomedical Sciences, Department of Immunology, Mayo Clinic, Rochester, Minnesota, USA

[b]Division of Clinical Microbiology, Department of Laboratory Medicine and Pathology, Mayo Clinic, Rochester, Minnesota, USA

[c]Department of Orthopedic Surgery, Mayo Clinic, Rochester, MN, USA

[d]Department of Quantitative Sciences, Mayo Clinic, Rochester MN, USA

[e]Division of Public Health, Infectious Diseases and Occupational Medicine, Department of Medicine, Mayo Clinic, Rochester, Minnesota, USA

#**CORRESPONDING AUTHOR:** Robin Patel, M.D.

Division of Clinical Microbiology, Mayo Clinic,

200 First Street SW, Rochester, MN 55905

507-538-0579

email: patel.robin@mayo.edu

Table S1. Clinical definitions of non-infected arthroplasty failure (NIAF) subtypes.

Subtype	Definition
Aseptic loosening	A spectrum of pain associated with radiographic evidence of progressive lucent lines, implant subsidence, or migration associated with manually loose components intraoperatively
Osteolysis	Mechanically induced bone loss identified as progressive zones of lucency on radiographs and pain with an associated trigger (polyethylene wear or failure or metallosis)
Instability	A broad categorization of both hip and knee arthroplasties that failed in the setting of recurrent total hip arthroplasty dislocations necessitating revision, or issues with flexion, mid-flexion, extension, or global instability of a total knee arthroplasty
Stiffness	A range of motion limitations leading to diminished function and/or necessitating manipulation under anesthesia or further revision; Also indicated as arthrofibrosis, or acquired idiopathic stiffness diagnoses
Other	Failure indications not fitting the above diagnoses

Table S2. Protein targets included in the Olink Proteomics Inflammation Panel.

4E-BP1	CXCL1	IL-17A	MMP-1
ADA	CXCL10	IL-17C	MMP-10
ARTN	CXCL11	IL18	NRTN
AXIN1	CXCL5	IL-18R1	NT-3
Beta-NGF	CXCL6	IL2	OPG
CASP-8	CXCL9	IL-20	OSM
CCL11	DNER	IL-20RA	PD-L1
CCL19	EN-RAGE	IL-22 RA1	SCF
CCL20	FGF-19	IL-24	SIRT2
CCL23	FGF-21	IL-2RB	SLAMF1
CCL25	FGF-23	IL33	ST1A1
CCL28	FGF-5	IL4	STAMBP
CCL3	Flt3L	IL5	TGF-alpha
CCL4	GDNF	IL6	TNF
CD244	HGF	IL7	TNFB
CD40	IFN-gamma	IL8	TNFRSF9
CD5	IL-1 alpha	LAP TGF-beta-1	TNFSF14
CD6	IL10	LIF	TRAIL
CD8A	IL-10RA	LIF-R	TRANCE
CDCP1	IL-10RB	MCP-1	TSLP
CSF-1	IL-12B	MCP-2	TWEAK
CST5	IL13	MCP-3	uPA
CX3CL1	IL-15RA	MCP-4	VEGFA

Table S3. Olink Proteomics Inflammation Panel proteins differentially expressed in periprosthetic joint infection (PJI) *versus* non-infected arthroplasty failure (NIAF) sonicate fluid samples, with associated receiver operative curve area under curve (AUC) values. Statistical significance was determined via Welch's 2-sample t-test, with Benjamini-Hochberg multiple comparison adjustment.

Protein Target	Log_2FoldChange	p-value	Adjusted p-value	AUC
Upregulated in PJI				
CCL20	3.05	<0.0001	<0.0001	0.87
OSM	2.48	<0.0001	<0.0001	0.85
EN-RAGE	2.61	<0.0001	<0.0001	0.84
IL6	2.27	<0.0001	<0.0001	0.83
IL-1 alpha	1.14	<0.0001	<0.0001	0.81
IL8	2.54	<0.0001	<0.0001	0.82
CXCL5	2.30	<0.0001	<0.0001	0.78
CXCL1	1.58	<0.0001	<0.0001	0.77
CXCL6	1.25	<0.0001	<0.0001	0.76
LIF	0.82	<0.0001	<0.0001	0.73
IL-17A	0.43	<0.0001	<0.0001	0.70
TNF	0.21	0.0003	0.0011	0.65
MMP-1	1.24	0.0007	0.0025	0.68
IFN-gamma	0.66	0.0014	0.0043	0.63
IL-18R1	0.50	0.0074	0.0199	0.61
CCL4	0.42	0.0110	0.0272	0.57
Downregulated in PJI				
CSF-1	-1.43	<0.0001	<0.0001	0.75
OPG	-1.42	<0.0001	<0.0001	0.75
Flt3L	-1.01	<0.0001	<0.0001	0.72
AXIN1	-0.36	<0.0001	<0.0001	0.70
TWEAK	-1.16	<0.0001	<0.0001	0.71
TNFRSF9	-1.05	<0.0001	<0.0001	0.71
MCP-1	-1.39	<0.0001	<0.0001	0.71
CDCP1	-0.59	<0.0001	<0.0001	0.71
SCF	-0.42	<0.0001	<0.0001	0.69
4E-BP1	-1.30	0.0001	0.0005	0.65
TRANCE	-0.63	0.0003	0.0011	0.67
CD40	-1.19	0.0003	0.0012	0.65
MMP-10	-0.67	0.0004	0.0016	0.65
ST1A1	-0.68	0.0008	0.0027	0.64
MCP-4	-0.84	0.0010	0.0034	0.62
IL18	-1.10	0.0021	0.0067	0.61
HGF	-0.78	0.0032	0.0096	0.63
IL-10RB	-0.13	0.0042	0.0121	0.62
CCL3	-0.74	0.0049	0.0136	0.63
STAMBP	-0.55	0.0098	0.0258	0.60
CXCL10	-0.61	0.0125	0.0302	0.61

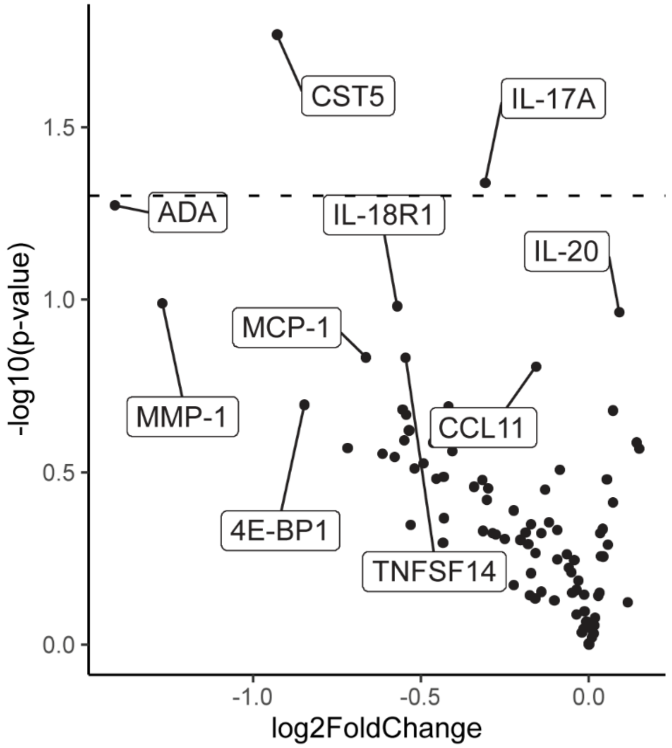

Fig S1. Volcano plot of Olink Proteomics Inflammation Panel proteins expressed in staphylococcal *versus* non-staphylococcal periprosthetic joint infection (PJI) sonicate fluid samples. No protein targets were statistically differentially expressed between the two groups as determined by Welch's 2-sample t-test, with Benjamini-Hochberg multiple comparison adjustment. The horizonal dashed line designates a p-value = 0.05.
